# The Impact of Food Service Attributes on Customer Satisfaction in a Rural University Campus Environment

**DOI:** 10.1155/2019/2154548

**Published:** 2019-12-27

**Authors:** Mireille Serhan, Carole Serhan

**Affiliations:** ^1^University of Balamand, Faculty of Health Sciences, Department of Nutritional Sciences, Deir El Balamand, P.O. Box 100, Tripoli, Lebanon; ^2^University of Balamand, Issam Fares Faculty of Technology, Department of Business Management and Administration, Deir El Balamand, P.O. Box 100, Tripoli, Lebanon

## Abstract

The purpose of this study was to determine different food service attributes that have an impact on customers' overall satisfaction at a rural university cafeteria. Over 5 weeks, 676 cafeteria users, including academics, staff, and students, were selected through convenience sampling. They completed an anonymous-designed survey with closed questions (*n* = 29) assessing quality of food and beverages, quality of service and setting, and satisfaction with food service attributes. In order to measure the existence and degree of significant relationships between different research variables, Pearson correlation coefficients were employed to analyse the data. Means of scores and frequencies were calculated. Results indicated that customers' satisfaction with different service attributes was above average. All service attributes had a significant and positive effect on the overall satisfaction. Since most customers (62.9%) would like to continue eating at the cafeteria, the most common improvements suggested to the university management included among others, improving diet quality by offering more nutritious food. Gaining insight into the different food service attributes can enable the university management to meet the needs and expectations of its academics, staff, and students in order to increase their confidence in the food provided.

## 1. Introduction

Cafeteria food services can be found in hospital facilities, nursing homes, child and senior care centers, prisons, schools, and university campuses. The quality of food service is one of the most relevant items of quality perceived by customers. In health care, the satisfaction of patients is ultimately related to the provided service quality [1]. In hotel restaurants, the quality of physical environment, service, and food affects guests' satisfaction and intention [2]. In the higher education milieu, more than ever, food service attributes have become an essential component affecting the quality of campus life [3, 4].

The majority of existing research on university food service has focused either on students' satisfaction with products, services, and service environments [3, 5–8] or on the nutritional intake of students consuming on campus food and their health implications [9–11].

Moreover, the higher education market has become competitive and global [12]. In this dynamic context, university food service operators have to adapt to changing expectations of their customers, increased competition from fast food segments on and off campus [13], and economic trends in uncertain markets [14]. According to Lugosi [15], when customers' expectations are high, the campus food services are expected to be more responsive. The workplace is a captive environment where the overall satisfaction of consumers could be an important element of the overall eating experience on campus [16].

**Figure 1:**
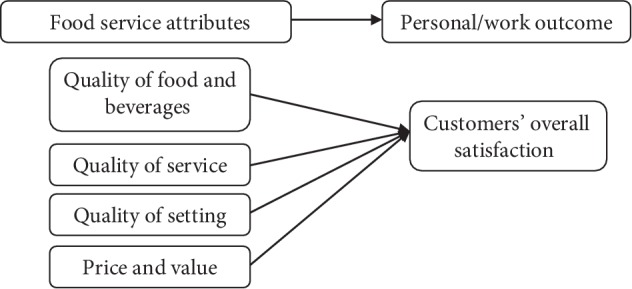
Food service attributes and customer satisfaction.

Therefore, building on previous research, the evaluation of university food services became essential. No previously published data investigated the quality of food service in Lebanese universities and its effect on customers' satisfaction, leaving a gap in the body of knowledge of costumers' opinions and behaviours of the on-campus food service in Lebanon. This study is aimed at addressing this issue through five main objectives: assess current opinion and explore the determinants of quality of food and beverages (1), service (2), setting (3), price and value (4), and the overall satisfaction of costumers (5) as presented in Figure 1. The study is also aimed at identifying future avenues for good practice that may inform facilities and service development decisions on what changes they would like to see to improve the on-campus food experience as part of constructive interventions.

## 2. Literature Review

### 2.1. Customers' Satisfaction in Higher Education

By reviewing the existing literature on customers' satisfaction, there are a large number of studies on customer's satisfaction in the private or public business sector. In the context of higher education, few studies on customer's satisfaction have been conducted [17–19]. According to Navarro and Iglesias [20], numerous attempts have been made by researchers to define the concept of satisfaction in relation to services offered in higher education [21–23]. They acknowledge that satisfaction is the final state of psychological process. Amelia and Garg [24] stated that the first impression is the one of the main considerations along with the quality and correctness of the served food and the gentleness of the staff in service. In university cafeterias, students make up the majority as users' satisfaction of institutional food services; thus, campus food service is becoming popular and important [3, 18, 19, 22]. Kwun [4] has taken into consideration the gender difference while studying the effect of campus food service attributes on perceived value, satisfaction, and consumer attitudes. According to Garg and Kumar [17], the dining experience has influenced the satisfaction and loyalty of both students and staff customers. In university cafeteria, customer satisfaction is totally related to the served food and beverage quality, variety and choices, to hygiene and cleanliness, and to price and value fairness [21, 25]. Based on the aforementioned attributes, there were many factors found to influence customers when choosing a food service.

### 2.2. Attribute 1: Quality of Food and Beverage

Previous studies indicated the degree of satisfaction with university cafeteria depends mostly on food and beverage quality [22, 26–28]. Food quality is the quality characteristics of food that is acceptable to customer [22]. Overall quality of the food and beverage, the taste, the freshness, the nutritious aspect, and the portion size is categorized under food quality measurement. As a core product of a food service operation, food and beverage quality has been given a great importance and has been checked for many aspects such as temperature, texture, flavour, and aroma [26–29]. Food and beverage quality is considered to affect the customers' intentions to come back again to a specific restaurant. Oh [23] found a high positive relationship between consumer satisfaction with food and beverage quality and their intention to continue eating in a specific restaurant. Furthermore, workplace eating is frequently associated with poor quality and bad food choices which have negative consequences [30]. Tam et al. [25] have stated various aspects for encouraging customers to eat healthy. Institutions have a responsibility to provide an environment that makes it easier for students to make healthier food easier. Previous research indicates that many institutions food environments are filled with energy-dense nutrient-poor food that may be heavily promoted [31, 32]. Moreover, it is the operators' role to provide a variety of products in their menus that will give its customers more options to choose from. The menu is definitely one of the key indicators of restaurants' marketing plans [33]. Accordingly, the following research hypothesis is thus posited:

#### 2.2.1. Hypothesis 1

Quality of food and beverage offered at university cafeteria has a significant and positive effect on customers' overall satisfaction.

### 2.3. Attribute 2: Quality of Service

Service quality is considered a key element in the restaurant sector, bearing in mind that dining in restaurants is essentially a social event [34, 35].

In some studies, it was found that service quality was more important than food quality in dining satisfaction. Yuksel and Yusel [36] suggested that service quality has significant effect on dining satisfaction at an aggregate market level and particularly for adventurous or healthy food seekers.

Furthermore, the quality of the service has been nowadays measured with respect to the customers' expectations and insights towards the offered service [37]. As per Inkumsah [38], it was found that customer satisfaction is affected by the quality of offered food service. In the same context, Garg [39] stated that food service has an impact on customers' perceptions towards a restaurant. Küçükaltan [40] declared that different customers can judge differently the same food service, and this is mainly related to the customers' opinions regarding the food service provided. If the offered service does not meet or is less than the customers' expectations, then the perceived service quality will be low; if it does exceed the customers' expectations, then the perceived service quality will be high [41]. Abo-Baker [42] described service quality as the organization's ability to satisfy the customers, within the determination of specifications, characteristics, and requirements of service that gratify the desires and needs of customers and exceed their expectations.

In the higher education milieu, according to Kim et al. [27], students' expectations and perceptions regarding the quality of service vary from one student to another and from one semester to the next. Hence, this variation leads to a more complex, diverse, and dynamic business environment, a difficulty in measuring service quality, and a difficulty in identifying the determinants of service quality. Tan et al. [43] specified that this intangible element is one of the vital components in service quality. Because services are intangible, it is difficult to measure them. Moreover, the employees especially in service quality play a vital role in the success of food service outlets. The personality traits and the use of social networking affect job satisfaction among workers [44].

Employees' behaviour affects customers' perceptions of service quality [45]. The interaction between cafeteria staff and customers, such as friendly gestures, e.g., greetings and high levels of responsiveness, cleanliness, and quick service, is important as it influences satisfaction with the service quality [46]. It is worth mentioning that service operators should enhance the quality of service provided on-campus to discourage students from searching for alternative food service operations off-campus. Students are not limited to on-campus food service quality, as they are aware of surrounding food service quality.

Many instruments were developed and refined by researchers for measuring perceived quality of service in the literature.

SERVQUAL is a known instrument which was implemented by Zeithaml et al. [47]. It consists of five service dimensions which are tangibles (physical facilities, equipment, and appearance of personnel), responsiveness, reliability, assurance, and empathy.

LODGSERV is another instrument, which was developed to assess service quality in hotels and function halls [45]. Additionally, Stevens et al. [48] adopted and refined the DINSERV scale from SERVQUAL and LODGSERV to assess customers' perceptions of restaurant quality. The DINSERV scale comprises 29 statements in five dimensions of the SERVQUAL scale. It is frequently used as a valid measurement tool to evaluate service quality in different hospitality establishments and mainly food service operations which is the case of the current study. Kim et al. [27] have investigated the relative importance of institutional DINESERV factors on customer satisfaction, return intention, and word-of-mouth in the university dining facility. Recently, service quality is influenced by the utilization of information technology, with reference to the signaling theory [49]. Accordingly, the following research hypothesis is thus posited:

#### 2.3.1. Hypothesis 2

Quality of service offered at university cafeteria has a significant and positive effect on customers' overall satisfaction.

### 2.4. Attribute 3: Quality of Setting

According to Kwun [4], the setting of the campus food service sampled is often referred to its environment and operational facets. The expectations and insights of customers differ based on where they consume. It is noteworthy to mention that the setting has been considered as a further dimension that has an impact on customers' insights towards campuses' food service. Several studies show that cleanliness, dining room environment, comfort level, operating hours and days, atmosphere, and capacity had significant effects on satisfactions and revisit intentions [26, 27, 50].

In a study conducted by Cardello et al. [51], home and traditional full service restaurants ranked higher than institutional food service, while airline and hospital food service ranked lower than school food service, with reference to the expected acceptability of quality of food.

Hence, prior research by Story et al. [52] found that food packaging, plate size and design, lighting, and dining companions at the cafeteria influences the individual's immediate setting.

The atmosphere is an intangible component made up of everything related to the brand that will yield an impression towards the specific location. The setting components can also include the seating's organization, the various decorations, and the music ambient [28]. Various scholars [53–55] identified a relationship among food information and quality, eating behaviours, seating's organization, and food distribution environment. Accordingly, the following research hypothesis is thus posited:

#### 2.4.1. Hypothesis 3

Quality of setting has a significant and positive effect on customers' overall satisfaction with the university cafeteria.

### 2.5. Attribute 4: Price and Value

In campus food service, it is noteworthy that students have restricted financial resources that influence their choices and decisions of picking food service operations, as they continually seek reasonable prices, due to limited budget [56]. Similarly, Nadzirah et al. [57] found that cost is the primary factor in university food service operations since students have limited funds. According to Nadzirah et al. [57], food service operators should ameliorate their menus through reconsidering their prices and thus ensuring customers are using the university cafeteria and not any off-campus food service operators. Soriano [58] found that the customers' quality expectations depend on the price they pay for receiving the service and when this price increases the quality expectations will increase consequently. In the same study, they showed that the price of a meal is equally important to other satisfaction determinants.

Several studies have been carried out by many researchers on price fairness or price and value. Price fairness means the judgment of whether an outcome or the process to reach an outcome is reasonable or acceptable [59]. In the same vein, the price to be paid for a service determines the level of quality to be demanded [58]. He also stressed that the price (value) of the meal and service are equally important when compared to other service dimensions. Ng [21] and Xi and Shuai [26] did consider price and value in assessing students' service quality in dining hall services. Martin-Consuegra et al. [60] found that perceived price fairness positively influences customer satisfaction. The effect of food quality, price fairness, staff performance, and ambience on students' satisfaction of cafeteria food services by comparing responses from two universities (MBU) was analysed using the partial least squares (PLS) application in Smart PLS computer software [61].

Similarly, Klassen et al. [50] found that price is the most significant factor in choosing a food and beverage service provider for students with limited budgets. In another study, customers indicated that receiving the right value for the money paid is among the most important factors that encourage them to revisit a food service establishment again [36]. Accordingly, the following hypothesis is posited:

#### 2.5.1. Hypothesis 4

Price and value have a significant and positive effect on customers' overall satisfaction with the university cafeteria.

## 3. Methodology

### 3.1. Research Approach and Sampling Method

The main aim of this study is to determine cafeteria customers' satisfaction and perceptions of quality of food and beverages and services offered at the university cafeteria. Therefore, in order to empirically test the suggested aforementioned hypotheses in this study, a quantitative research approach, based on the distribution of personally administered questionnaires, was the applied method, allowing respondents to have more time to complete the questionnaire and making it easier and more convenient for them to respond. It involves the collection of customer-based data, which can be analysed statistically [62]. The target population of this research study included all academics, staff, and students at a rural university in Lebanon. According to official data pertaining to the university for academic year 2018-2019, there are more than 6,000 academics, staff, and students. Due to this large number, it is difficult to use random sampling techniques. Therefore, a convenience sampling technique is the most suitable sampling technique to use in this research.

With reference to the new management body of the university, it is working effectively through different approaches to improve student retention. These approaches include identifying and prioritizing the main reasons for student recruitment and corresponding retention solutions. The new management body of the university has taken the initiative to involve students in the decision-making process about food services, as well as in many other academic/service areas. The management body requested that there should be a process by which the university cafeteria operator will be continuously evaluated; students and other customers will have an input in evaluating the food services on campus. The new management body of the university will monitor the improvement actions for the coming years to measure their efficiency based on student feedback and to identify areas warranting further improvement attention.

### 3.2. Survey Development

The questionnaire in the current study was adopted from a previously validated tool used by El-Said and Fathy [3], with modifications. In comparison with El-Said and Fathy [3], the sample includes more categories (academics and staff), in order to provide more representative results and to improve sample generalizability. It comprised two sections. The first section is aimed at collecting demographic data of cafeteria customers and their behaviour characteristics (academic, staff, student, gender, and age; number of visits to the cafeteria, monthly expenditure, and intention to continue eating at the cafeteria). The second section of the questionnaire consisted of four parts. Statements in these parts were adapted from the DINESERV questionnaire. DINESERV is adapted from the SERVQUAL instrument and was created by Barsky [46] and designed for the food service industry. The first part of the second section consisted of eight statements and aimed at measuring customers' perceptions of quality of food and beverages offered at the cafeteria. Part two of the second section consisted of 4 statements and aimed at measuring customers' service quality perceptions in the cafeteria. Part three of the second section consisted of 5 statements and aimed at measuring customers' perceptions regarding the quality of the setting. The fourth part of the second section was designed to measure customers' perceptions of price compared to the value they receive. The questionnaire of the last section is aimed at measuring customers' overall satisfaction in terms of overall satisfaction with food and beverage quality, overall satisfaction with service quality, overall satisfaction with the quality of the setting, overall satisfaction with the price paid versus the value obtained, and their overall satisfaction with the dining experience. A 5-point Likert scale will be used for evaluation, where 5 = very satisfied, 4 = satisfied, 3 = neutral, 2 = unsatisfied, and 1 = very unsatisfied.

In order to determine the internal consistency of the survey questionnaire, a Cronbach's alpha coefficient reliability analysis was performed. This method shows an indication of the average correlation between all the items of the research questionnaire on the Likert scale, in this case. The Cronbach's alpha coefficient for the questionnaire was measured to be 0.960. Therefore, the Cronbach's alpha coefficient is well above the 0.7 standard reliability. Item analysis was achieved as well to provide item-to-total correlations and Cronbach's alpha if the item was deleted from the survey questionnaire. To evaluate the construct validity, exploratory factor analysis (EFA) with promax rotation was conducted. Finally, to check the content validity, a convenience sample of panel of experts (6 professors who were familiar with the scope of the study) checked the questionnaire through reviewing the content of each item in the modified version. Results showed that the final version of the questionnaire is valid and reliable and can be used in future studies for testing customers' satisfaction and perceptions of quality of food and beverages and services offered at university cafeterias.

### 3.3. Implementation and Participants

Before implementation, the survey was piloted to 30 persons (5 academics, 10 staff, and 15 students) to discover the extent of their understanding of sentences as well as the time taken to answer questions. Finally, based on the pilot test review, minor changes were performed to reach the final version of the questionnaire.

In order to calculate the sample size, there is a need to determine the accurate population size, the margin of error, and the confidence level. The most common used margin of error is 5% and the most common used confidence level is 95%. These percentages are standards in quantitative research [63]. Using the G∗Power sample size software, version 3.1.3 (http://www.psycho.uni-duesseldorf.de/abteilungen/aap/gpower3/, Faul et al. [64]), one of the leading software used for sample size calculation in various fields, a minimum of 362 respondents were required to achieve power for a population of 6000 based on precision level of 5%, confidence internal level of 95%, and *P* = 0.05.

To guarantee the collection of the targeted sample size, students as part of their work on campus, students were asked to help in the data collection and given information about the research topic and the content of the survey form. Additionally, they were trained on how to deal with respondents and how to gather required data. They approached their peers, as well as academics and staff from different faculties and asked them in person to fill out the questionnaire. It took between 5 and 10 minutes to complete. Anonymity was ensured. A total of 676 questionnaires were collected during the period of December 2018-January 2019. From this number, 24 questionnaires were invalid, and therefore, the total target sample for this study was 652 freshmen, undergraduate, and graduate students, academics, and professional staff employed at this rural university. The latter has an on-campus food service venue with 150 seats.

### 3.4. Statistical Analysis

Only completed surveys were analyzed. Data was processed and analyzed by the SPSS statistical software, Windows Version 23.0 (SPSS, Inc., Chicago, IL, USA). Means and frequencies as well as coefficients of Pearson correlations were used, in order to achieve the objective of this study. Frequencies were computed to examine demographic and behavioural characteristics of respondents. Means of scores were calculated in order to assess students' perceptions regarding different service attributes.

### 3.5. Ethical Considerations

For ethical considerations, a written permission to use and modify the survey questionnaire was obtained by the authors of the original questionnaire [3]. Ethical approval to conduct the study and to contact academics, staff, and students was obtained from the University Management Board. An informed consent was signed by those who agreed to fill the questionnaire.

## 4. Results

### 4.1. Demographic and Behavioural Characteristics of Respondents

The demographic characteristics of respondents are presented in Table 1.

The sample consisted of 37.4% male respondents and 62.6% female respondents. Among the 652 respondents, 83.7% of respondents were university students, 10.9% were academic, and 5.4% were staff.

A high percentage of respondents (62.0%) were aged between 17 and 21 years, 22.0% were between 22 and 24 years old, only 6.60% were between 25 and 35, and 9.4% were 35 years old and above.

Table 2 shows the behavioural characteristics of respondents. As shown in the table, 3.5% of surveyed respondents visited the cafeteria daily, 13.4% visited the cafeteria twice a week, and 26.7% of respondents visited the cafeteria once a week.

Approximately half of the respondents (56.4%) visited the cafeteria once a month.

Only 8.3% of respondents reported that their monthly average expenditure was above 100 000 Lebanese Pounds, and approximately half of the respondents (48.5%) spent between 10 000 and 50 000 Lebanese Pounds monthly. Furthermore, about 37.1% of respondents did not intend to continue having their meals at the university cafeteria, while almost the majority (62.9%) would like to continue eating at the cafeteria.

### 4.2. Food Service Attributes and Customer Satisfaction

The means of scores of respondents' perceptions of different research variables were computed, as presented in Table 3. Respondents rated their levels of satisfaction with attitude statements that were positively phrased using a scale from 1 to 5, with 1 = very unsatisfied and 5 = very satisfied. Firstly, respondents' overall perceptions regarding the quality of food and beverage products presented at the cafeteria were above average (overall mean for the quality of food and beverage items = 3.41). According to the results presented in the table, a high percentage of respondents were satisfied with the taste of food and beverages (*M* = 3.46), as well the display (*M* = 3.45) and diversity of products (*M* = 3.42). Respondents satisfaction with the freshness of food and beverage items (*M* = 3.39), the nutritious products (*M* = 3.34), and the appropriate serving temperature (M = 3.39) recorded the lowest mean score among the quality attributes. Similar opinions were given about the items related to the quality of service. Respondents' overall perceptions regarding the service quality presented at the university cafeteria were above the average (overall mean for the service quality attributes = 3.53). The friendly treatment by cafeteria staff, the staff knowledge of the items sold, and the cooperation of workers recorded the highest mean score among service quality attributes. Satisfaction means ranged from 3.57 to 3.61. However, the speed of service recorded the lowest mean score (*M* = 3.46).

The third variable that respondents were asked about was the quality of the setting. The ambience, the lighting, and the organization of the delivery process recorded the highest mean score, above the mean (*M* = 3.31). The cleanliness and hygiene (*M* = 3.18) as well as the comfort and sitting availability (*M* = 3.26) recorded the lowest mean score.

Opinions were given about the price respondents paid compared to the value they received. The value that respondents received was measured in terms of the quality and quantity of food and beverage items they received. As shown in Table 3, most respondents felt that the quantity of food and beverage items provided was suitable and above the mean score, given the price paid (*M* = 3.25). Additionally, respondents' satisfaction with the quality of food and beverage items, given the price paid, was perceived to be not satisfactory (*M* = 3.20).

The last research variable measured was respondents' overall satisfaction. Overall respondents' satisfaction was measured using the following statements: overall satisfaction regarding the quality of food and beverage items (*M* = 3.42) and overall satisfaction regarding the service quality (*M* = 3.51) recorded the highest mean score, above the mean (*M* = 3.39). Overall satisfaction regarding the prices (*M* = 3.24) and overall satisfaction regarding the setting (*M* = 3.38) recorded the lowest mean score.

As shown in Table 4, the existence and level of correlation between different research variables and respondents' overall satisfaction were investigated using the Pearson correlation coefficient. The results indicated a significant correlation between food and beverage quality and respondents' overall satisfaction (*r* = 0.873, *P* < 0.01). The Pearson correlation coefficient values emphasize the positive correlation between food and beverage quality and students' overall satisfaction. Therefore, H1 was supported after the Pearson correlation testing was performed.

Furthermore, the results of the Pearson correlation test revealed a significant and positive correlation between service quality (*r* = 0.834, *P* < 0.01), setting quality (*r* = 0.836, *P* < 0.01), and respondents' overall satisfaction (*r* = 0.959, *P* < 0.01). Therefore, the resulting hypotheses H2 and H3 were also supported. Results indicated that there was a statistically significant and positive association between the price and value (*r* = 0.853, *P* < 0.01) and respondents' overall satisfaction, with reference to H4.

## 5. Discussions and Conclusions

The purpose of this study was to determine the cafeteria customers' overall satisfaction with on-campus food service attributes. The findings suggest some important implications for university food service operator. The food service manager should recognize the customers' characteristics such as age groups. The results of the study showed that the age groups between 17 and 21 are the largest customers. Therefore, the campus food service manager should develop strategies catered to appeal different segments of customers based on the various age groups.

The regression analysis showed that the quality of service was the strongest predictor of customer satisfaction. Thus, university food service operator should continue to train their employees to greet their customers in a polite manner, to be attentive and friendly, and to increase their knowledge about the food items served. Maintaining the quality of their service ensures that they can still continue to meet or exceed costumer expectations [65]. Lashley [66] has shown that sincere and affective relationships between the host and the guest can operate in a commercial environment.

Food and beverages quality turned out to be the second important element affecting customer satisfaction. In sum, some of the possible strategic implementations may include more variety of nutritious products, adjusting the serving temperature, and paying more attention to the freshness of the products sold. This result is consistent with the previous findings of Kjøllesdal et al. [30]. Kjøllesdal et al. [30] asserted that workplace eating is frequently associated with poor-quality food and bad choices, which have negative consequences. In rural universities, accessing food in places of work, as healthy options and varied choices may be limited. Ham [8] mentioned that good-quality food service provision can contribute to the overall campus experience. Absence of trust in the quality of food has an impact on diet through avoidance of certain products deemed to be unsafe or untrustworthy [67]. The challenge for the university food service operator is to provide products and services that enhance and facilitate positive healthy food choices. Given the amount of employees eating at their place of work, most research on this topic relates to the direct importance of making healthy dishes available [68].

Furthermore, the university food service operator should pay more attention to the quality of the setting. They should carefully design cafeteria interiors and exteriors to deliver a relaxed and comfortable atmosphere to attract new customers and to retain current ones. University food service operator should maintain the cleanliness and hygiene of the facility to a standard level. The findings are in line with the previous results of Kim et al. [7]. Improving customer satisfaction with reference to the quality of the setting will not only strengthen the customer loyalty but also improve the facility reputation and this is also good for their businesses. Lugosi [15] has studied the campus food service experience with reference to student well-being and has emphasized on the campus food service as a cowork space. Among several factors driving social interaction, contemporary designs of university campuses have adopted many of the features of cowork spaces [69–71], with furnishings and layout of the infrastructure of the space, facilitating the positive experience.

Particularly, cleanliness or hygiene was the third most important factor, after food variety and convenient location, which influences costumer selection of a food service to dine in. Although costumers are increasingly concerned about the nutritional value of the food they consume, food safety remains far more important than as the associated risk can be substantial. Food service hygiene is indeed important. Fatimah et al. [72], in their study, have identified four underlying food service hygiene factors from the consumer perspective: food and location, staff and handling, premise and practices, and ambient scent. The priority should be given to service quality. Low service quality is attributed to low-scale food services.

Moreover, customers tend out to be the least dissatisfied with the price paid, with reference to the quality of food and beverage products provided. The university food service operator should improve the quality of the products served and should offer reasonable pricing, in order to prevent customers from switching to other off-campus restaurants, which will result in less sales and lower revenue in the long term. Higher customer satisfaction should increase revisit/return intention and provide word-of-mouth endorsements of the university food service facility [73].

From the managerial perspective, the great importance of customer place on the quality of the food service requires that the food service provided by the university campus should take into consideration the customers' insights and perceptions and thus give a push to many institutions to overhaul their campus food service operations. Demand for healthy food and quality of the setting, with reference to the comfort of the sitting area, is an important lever for positive and promising change.

## 6. Limitations and Future Research

The limitations of the study are that a single university campus cannot represent all the university campuses and all universities in Lebanon. Results should be interpreted with caution. Also, the survey questionnaire was distributed by students. This might affect students' attitudes and opinions as they took the survey. For future research, it would be important to replicate the study on another campus, to determine how and if the findings hold true given a diverse sample, in an urban campus. Another constraint of this research is the feature of its samples. More than 80% of the participants in the survey were students. Surprisingly, the majority of staff and academics were not interested in filling out the survey. Therefore, performing another study in a larger scale is suggested to expand the results of this research and to provide more representative results and to improve sample generalizability. The current study can, however, help to provide a roadmap for helping the university management better understand the key importance of food and service quality. Based on the results, several implications and recommendations could be derived for university management to increase student satisfaction about food and beverage services provided by university cafeteria. University management (1) should investigate about cafeteria users' opinions continuously in order to solve any problems promptly, (2) should institutionalize systems for continuous training of cafeteria employees through customized programs designed for them, (3) should invest in improving the quality of the setting, with reference to the comfort of the sitting area, (4) should invest, in coordination with the cafeteria operator, in offering more nutritious food in order to be able to meet cafeteria users' needs, (5) should give special attention to contract with the best operator, (6) should develop strategies catered to appeal different segments of customers based on the various age groups, and (7) should place more emphasis on identifying and meeting the needs of students and staff (offering late night meals).

## Figures and Tables

**Table 1:** Demographic characteristics of respondents.

Demographics	Frequency	Percentage (%)
*Gender*		
Male	244	37.4
Female	408	62.6
*Age*		
17–21	404	62.0
22–24	143	21.9
25–35	43	6.6
35 and above	62	9.5
*Cafeteria users*' *status*		
Academic	71	10.9
Staff	35	5.4
University student	546	83.7

**Table 2:** Behavior characteristics of respondents.

Behavior characteristics	Frequency	Percentage (%)
*Average number of visits to the cafeteria*		
Once/month	368	56.4
Once/week	174	26.7
Twice/week	87	13.4
Daily	23	3.5
*Monthly Average Expenditure in Lebanese Pounds* (*LBP*)^∗^		
Less than 10,000	226	34.7
10,000–50,000	316	48.5
50,001–100,000	56	8.6
More than 100,000	54	8.3
*Intention to continue eating at the cafeteria*		
Yes	410	62.9
No	242	37.1

^∗^1 LBP = 0.00066 USD.

**Table 3:** Frequencies and means for the research variables.

Research variables	Overall satisfaction										Mean
	Very unsatisfied		Unsatisfied		Neutral		Satisfied		Very satisfied		
	*F*	%	*F*	%	*F*	%	*F*	%	*F*	%	
*A. Quality of food and beverage products*											
(1) Taste of food and beverages	20	3.1	48	7.4	236	36.2	306	46.9	42	6.4	3.46
(2) Display of products	16	2.5	64	9.8	212	32.5	326	50.0	34	5.2	3.45
(3) Diversity of displayed products	16	2.5	74	11.3	218	33.4	306	46.9	38	5.8	3.42
(4) Freshness of products	28	4.3	64	9.8	230	35.3	284	43.6	46	7.1	3.39
(5) Taste and flavor of products	20	3.1	58	8.9	230	35.3	310	47.5	34	5.2	3.42
(6) Nutritious products	32	4.9	62	9.5	250	38.3	266	40.8	42	6.4	3.34
(7) Portion size	30	4.6	70	10.7	208	31.9	288	44.2	56	8.6	3.41
(8) Appropriate serving temperature	20	3.1	72	11.0	224	34.4	300	46.0	36	5.5	3.39
Overall mean for quality of food and beverage products											3.41
*B. Quality of service*											
(1) Speed of service	38	5.8	90	13.8	188	28.8	280	42.9	56	8.6	3.46
(2) Friendly treatment by the cafeteria staff	24	3.7	68	10.4	174	26.7	282	43.3	104	16.0	3.57
(3) Staff knowledge of the items sold	24	3.7	38	5.8	190	29.1	312	47.9	88	13.5	3.61
(4) Cooperation of workers at the cafeteria and answering any question	30	4.6	42	6.4	174	26.7	320	49.1	86	13.2	3.59
Overall mean for service											3.53
*C. Quality of the setting*											
(1) Comfort and sitting availability	36	5.5	76	11.7	250	38.3	260	39.9	30	4.6	3.26
(2) Cleanliness and hygiene	28	4.3	102	15.6	276	42.3	214	32.8	32	4.9	3.18
(3) Ambience	22	3.4	64	9.8	274	42.0	258	39.6	34	5.2	3.33
(4) Lighting	16	2.5	44	6.7	258	39.6	290	44.5	44	6.7	3.46
(5) Organization of Delivery Process	32	4.9	54	8.3	256	39.3	272	41.7	38	5.8	3.35
Overall mean for setting											3.31
*D. Price and value*											
(1) Quality of food and beverage products provided vs. the price paid	36	5.5	108	16.6	226	34.7	248	38.0	34	5.2	3.20
(2) Quantity of food and beverage products provided vs. the price paid	26	4.0	100	15.3	244	37.4	248	38.0	34	5.2	3.25
Overall mean for price and value											3.23
*E. Overall satisfaction*											
(1) Overall satisfaction regarding the quality of food & beverage products	26	4.0	60	9.2	214	32.8	312	47.9	40	6.1	3.42
(2) Overall satisfaction regarding the service quality	32	4.9	52	8.0	182	27.9	318	48.8	68	10.4	3.51
(3) Overall satisfaction regarding the setting	24	3.7	44	6.7	276	42.3	270	41.4	38	5.8	3.38
(4) Overall satisfaction the prices	32	4.9	88	13.5	254	39.0	244	37.4	34	5.2	3.24
Overall mean for overall satisfaction											3.39

**Table 4:** Variables' correlations.

		Quality of food and beverages	Quality of service	Quality of setting	Price and value	Overall satisfaction	Combined effect of research variables
Quality of food and beverages	Pearson correlation		.622^∗∗^	.651^∗∗^	.729^∗∗^	.831^∗∗^	.873^∗∗^
	Sig. (2-tailed)		.000	.000	.000	.000	.000
	*N*		652	652	652	652	652

Quality of service	Pearson correlation	.622^∗∗^		.669^∗∗^	.552^∗∗^	.765^∗∗^	.834^∗∗^
	Sig. (2-tailed)	.000		.000	.000	.000	.000
	*N*	652		652	652	652	652

Quality of setting	Pearson correlation	.651^∗∗^	.669^∗∗^		.582^∗∗^	.768^∗∗^	.836^∗∗^
	Sig. (2-tailed)	.000	.000		.000	.000	.000
	*N*	652	652		652	652	652

Price and value	Pearson correlation	.729^∗∗^	.552^∗∗^	.582^∗∗^		.815^∗∗^	.853^∗∗^
	Sig. (2-tailed)	.000	.000	.000		.000	.000
	*N*	652	652	652		652	652

Overall satisfaction	Pearson correlation	.831^∗∗^	.765^∗∗^	.768^∗∗^	.815^∗∗^		.959^∗∗^
	Sig. (2-tailed)	.000	.000	.000	.000		.000
	*N*	652	652	652	652		326

Combined effect of research variables	Pearson correlation	.873^∗∗^	.834^∗∗^	.836^∗∗^	.853^∗∗^	.959^∗∗^	
	Sig. (2-tailed)	.000	.000	.000	.000	.000	
	*N*	652	652	652	652	326	

^∗∗^Correlation is significant at the 0.01 level (2-tailed).

## Data Availability

The data used to support the findings of this study are included within the article.
